# iCardio: The Brazilian Population-Based Real-World Data Platform for Cardiovascular Disease

**DOI:** 10.1016/j.mcpdig.2025.100255

**Published:** 2025-07-28

**Authors:** Miriam Allein Zago Marcolino, Ana Paula Beck da Silva Etges, Luciana Rodrigues de Lara, Nayê Balzan Schneider, Yohan Casiraghi, Wanderson Maia Da Silva, Carisi Anne Polanczyk

**Affiliations:** a National Institute of Science and Technology for Health Technology Assessment (IATS)—CNPq/Brazil (project: 465518/2014-1), Universidade Federal do Rio Grande do Sul (UFRGS), Porto Alegre, Brazil; b Postgraduate Program in Epidemiology, Universidade Federal do Rio Grande do Sul, Porto Alegre, RS, Brazil; c Postgraduate Program in Cardiology, Universidade Federal do Rio Grande do Sul, Porto Alegre, RS, Brazil; d Medical School, Universidade Federal do Rio Grande do Sul, Brazil

## Abstract

Technological advances that contribute to improving organizations and systems’ capability to manage care services and pathways are impactful in improving efficiency and reducing waste in health care. This narrative paper presents the implementation of iCardio, a dashboard of population real-world data-based analytical online open-access solution for the cardiovascular field in Brazil. The platform was developed using hospitalization data from patients who underwent cardiovascular operation or interventional procedures, identified by procedure codes reimbursed by the public health system. Patient-level data from hospital and mortality systems were provided by the Brazilian Ministry of Health, cleaned, and organized into individual-level and hospitalization-level datasets to enable parameter calculation. A web-based solution was developed to provide user-friendly, interactive access to 17 indicators relevant to evaluating cardiovascular service efficiency, quality, and equity. Data from 291,490 patients with 317,338 index hospitalizations and 375,809 procedures (172,874 of cardiovascular operations and 202,935 of interventional cardiology) performed in 558 health care centers in Brazil compose the dataset behind the platform. The platform offers 4 analytical views: “patients,’ profile,’’ “by location,’’ “procedure rates,’’ and “detailed exploration,’’ displaying data by year (2019-2020) with multiple stratification options (eg, patient characteristics, procedures, health care centers, and geography). The iCardio is an online open-access platform based on real-world data that provides ready-to-use information about cardiovascular care in Brazil, which can be used as a transformative tool to sustain data-driven health policies and research in the cardiovascular field in Brazil.

Brazil has the most extensive universal health care system, with 49 nonintegrated health care-related databases.^1^ The registers of care services delivered are based at a procedure level, requiring computational techniques to use the available data to identify center, care pathways, and patient-level information that policymakers can better use to sustain resource allocation and health policies.^2^ The design and development of Business Intelligence tools in the health care sector have become increasingly prominent, offering the advantage of enabling graphical, dynamic, and interactive visualization of collected data and allowing real-time updates.^3^

Cardiovascular diseases are the number one cause of morbidity and of mortality worldwide and have a relevant impact on the financial budget, such that effective use of available resources is of utmost importance in universal health systems such as the Brazilian National Health System (Sistema Único de Saúde [SUS]).^4^ Real-world data (RWD) initiatives that provide information to measure access, quality of care, outcomes, and resource use related to cardiovascular diseases are fundamental to establish more assertive resource management processes and to reduce inequity.

In this context, this study reports a population RWD-based analytical solution for the cardiovascular field in Brazil (iCardio: dashboard of Brazil’s universal health care system cardiovascular care indicators), developed to turn information available and user-friendly to the general society.

### Approach

The iCardio development process was divided into the following 4 phases: (i) defining the indicators that matter for evaluating the efficiency and quality of cardiovascular services; (ii) accessing individual-patient level data from the public national databases; (iii) cleaning and structuring the data; and (iv) designing and coding the web analytical solution.

(i) Defining the indicators that matter for evaluating the efficiency and quality of cardiovascular services

The indicators were defined based on the opinions of cardiology experts and a data analyst who knew the data available in the data sources during the project development. Various indicators were proposed, and the final selection included clinical, care process, costs, access, and performance indicators to be assessed at national, regional, state, municipal, and center levels.

(ii) Accessing individual-patient level data from the public national databases

the Hospital Information System (Sistema de Informação Hospitalar [SIH]) and Mortality Information System (Sistema de Informação sobre Mortalidade [SIM]) were the sources for the iCardio development. Both databases included data of patients treated by the Brazilian Public Health System (Sistema Único de Saúde [SUS]).

The SIH database is composed of hospital admission authorization (Autorização de Internação Hospitalar [AIH]) records, which compiles hospital-related information and the procedures billed to the Ministry of Health. All procedures are recorded following standard procedure codes covered by the Brazilian Public Health System (SUS). A single hospitalization can be composed of a single or multiple AIH records, depending on the procedures to be billed and other admission characteristics.

The SIM database is composed of all death certificates emitted in the country, which includes information about the date and characteristics of the death, including cause and circumstance.

These information systems were originally independent, but a national patient-level database was created through a probabilistic linkage process managed by the Department of Informatics of the Brazilian National Health System (Departamento de Informática do SUS).^2^ The Ministry of Health granted direct access to this database to develop this project, including data from January 2019 to March 2021 from the SIH and January 2019 to October 2020 from the SIM.

(iii) Cleaning and structuring the data

After an exploratory analysis of the databases, a filter was applied to select the eligible patients. All hospitalization data from patients submitted to a cardiovascular operation or an interventional cardiovascular procedure in the period were selected based on the procedure code starting with Aril 6, 2021 or April 6. 2023,^5^ respectively, in any hospitalization. Cardiovascular operation involves surgical interventions on the heart and great vessels. Examples include atrial septal defect closure, aortic valve replacement, and coronary artery bypass grafting. Interventional cardiovascular procedures include minimally invasive techniques such as percutaneous atrial septal defect closure, coronary angioplasty with stent placement, and percutaneous aortic valvuloplasty. After this filter, preprocessing was performed to clean, standardize, and classify the data. Using the individual-patient code (ID), AIH registers were compiled into hospitalizations (including all sequential or superposed AIHs from the same patient at the same health care center) and then classified by the presence of a procedure of interest as index hospitalizations or not (whether no procedure of interest was identified). The follow-up of each patient started at the first index hospitalization until 30 days after the closure date of the last index hospitalization. All individual cardiovascular procedures were considered within an index hospitalization.

The SIH data were linked to the SIM data using the patient's ID. Due to the linkage nature of the patients' ID, a few inconsistencies had to be corrected, including readmissions after death, sex, race/color, age, and residency municipality. Any hospitalization that was registered after the death date was removed from the database, as were hospitalizations ending 2 or more days after the death register date. For patients without a death register but with death recorded in the hospitalization, the last hospitalization day was used as the death date.

Each index hospitalization was used as a starting point of follow-up for calculating the indicators. Other hospitalizations were used to calculate readmissions occurring within 30 days of the procedure or 30 days of the discharge of an index hospitalization. The patients’ travel distance to the procedure was estimated using the residency and center municipalities’ georeferencing (latitude and longitude) and calculated as the geodesic between these points and converted into kilometers.

The database was then structured by patient and index hospitalization and included only variables used for the indicators. All data processing was performed in R language (version 4.2.0) using the RStudio (version 2023.03.1 Build 446, Posit Software, PBC) for Windows.

(iv) Designing and coding the web analytical solution

Further data preparation for the dashboard was performed using Python (version 3.11.1) and Jupyther Notebook (version 3.5.3). The dashboard was developed in Microsoft Power Business Intelligence (version 2.124.2028.0).

The Institutional Research and Ethics Committee of Hospital de Clínicas de Porto Alegre approved the study, project number CAAE 66784523.0.0000.5327, and all data processing followed the national data protection law.

### Findings

The iCardio is composed of 17 leading indicators, described in Table 1. Each indicator can be analyzed based on multiple stratification factors, including patients' characteristics, specific procedures, health care centers, geographic location, and hospitalization year.

**Table 1:** iCardio Main Indicators

Indicator	Formula	Information Provided
Total patients	$\sum {Patients\;}_{V\left(i\right)\cap V\left(j\right)V\left(k\right)\cdots V\left(z\right)}$	Total number of patients who underwent at least one cardiovascular procedure. These data can be used to describe the profile of the patients, including aspects such as age distribution, sex, and geographic origin, based on the applied stratification filters.
Total procedures	$\sum {Procedures\;}_{V\left(i\right)\cap V\left(j\right)V\left(k\right)\cdots V\left(z\right)}$	Total of procedures performed, by stratification factors applied.
Total hospitalizations	$\sum {Hospitalizations\;}_{V\left(i\right)\cap V\left(j\right)V\left(k\right)\cdots V\left(z\right)}$	Total of index hospitalizations, by stratification factors, applied.
Mean LOS (d)	${\underline{X}\;Hospitalization\;days\;}_{V\left(i\right)\cap V\left(j\right)V\left(k\right)\cdots V\left(z\right)}$	The mean length of stay for an index hospitalization is calculated by the difference between the admission and discharge dates, taking into account the applied stratification factors.
Total expenses (R$)	$\sum {Payment\;per\;case\;}_{V\left(i\right)\cap V\left(j\right)V\left(k\right)\cdots V\left(z\right)}$	Total of payment for all index hospitalizations, by stratification factors applied.
Mean payment per case (R$)	${\underline{X}\;Payment\;per\;case\;}_{V\left(i\right)\cap V\left(j\right)V\left(k\right)\cdots V\left(z\right)}$	Mean payment by index hospitalization, by stratification factors applied.
ICU admission (%)	${\left(\frac{\sum Hospitalizations\;with\;ICU\;admission}{\sum Hospitalizations}\right)}_{V\left(i\right)\cap V\left(j\right)V\left(k\right)\cdots V\left(z\right)}$×100	Proportion of index hospitalizations with admission to an ICU, by stratification factors applied.
Mean ICU LOS (d)	${\underline{X}\;ICU\;days\;}_{V\left(i\right)\cap V\left(j\right)V\left(k\right)\cdots V\left(z\right)}$	Mean length of stay in ICU of index hospitalizations, by stratification factors applied.
Mean travel distance to health care center (Km)	${\underline{X}\;Travel\;distance\;to\;center\;}_{V\left(i\right)\cap V\left(j\right)V\left(k\right)\cdots V\left(z\right)}$	Mean distance (geodesic) in kilometers from the patient’s residency municipality to the health care center of an index hospitalization, by stratification factors applied.
Readmission within 30 d of the procedure (%)	${\left(\frac{\sum Readmissions\;in\;the\;period}{\sum Hospitalizations}\right)}_{V\left(i\right)\cap V\left(j\right)V\left(k\right)\cdots V\left(z\right)}\times 100$	Proportion of index hospitalizations with a readmission for any cause occurring within 30 d of the procedure date, by stratification factors applied.
Readmission within 30 d of the discharge (%)	${\left(\frac{\sum Readmissions\;in\;the\;period}{\sum Hospitalizations}\right)}_{V\left(i\right)\cap V\left(j\right)V\left(k\right)\cdots V\left(z\right)}\times 100$	Proportion of index hospitalizations with a readmission for any cause occurring within 30 d of the discharge date, by stratification factors applied.
In-hospital deaths (%)	${\left(\frac{\sum Deaths\;during\;hospitalization}{\sum Hospitalizations}\right)}_{V\left(i\right)\cap V\left(j\right)V\left(k\right)\cdots V\left(z\right)}\times 100$	Proportion of the index hospitalizations with death by any cause before the closure date, by stratification factors applied.
Deaths within 30 d of the procedure (%)	${\left(\frac{\sum Deaths\;in\;the\;period}{\sum Hospitalizations}\right)}_{V\left(i\right)\cap V\left(j\right)V\left(k\right)\cdots V\left(z\right)}\times 100$	Proportion of the index hospitalizations with death within 30 d of the procedure, by stratification factors applied.
Deaths within 30 d of the discharge (%)	${\left(\frac{\sum Deaths\;in\;the\;period}{\sum Hospitalizations}\right)}_{V\left(i\right)\cap V\left(j\right)V\left(k\right)\cdots V\left(z\right)}\times 100$	Proportion of the index hospitalizations with death within 30 d of the discharge, by stratification factors applied.
Type of procedure performed (%)	${\left(\frac{\sum Procedures\;by\;classification}{\sum Procedures}\right)}_{V\left(i\right)\cap V\left(j\right)V\left(k\right)\cdots V\left(z\right)}\times 100$	Proportion of the procedures related to a defined classification (procedure code, group, or organization), by stratification factors applied.
Crude procedure rate by million population	${\left(\frac{\sum Procedures\;by\;classification}{\sum Local\;population}\right)}_{V\left(i\right)\cap V\left(j\right)V\left(k\right)\cdots V\left(z\right)}$×1,000,000	Ratio between the total number of procedures referring to a specific classification (such as procedure code, group, or organization), and the local population defined by the residency of the patients, by stratification factors applied.
Age and sex standardized procedure rate by million population	${\left(\frac{\sum Procedures\;by\;classification}{\sum Standard\;population}\right)}_{V\left(i\right)\cap V\left(j\right)V\left(k\right)\cdots V\left(z\right)}$×1,000,000	Ratio between the expected total of procedures, referring to a defined classification (such as procedure code, group, or organization), assuming that the local population follows the age and sex distribution of the standard population (Brazilian 2020 population), by stratification factors applied.

Abbreviations: ICU, intensive care unit; Km, kilometers; LOS, length of stay; R$, Brazilian Reais; ∑, Sum; $\underline{X,}$ Mean; ∩, Intersection; $V_{\left(i\right)}...V_{\left(z\right),}$ Stratification variables available in the dashboard.

The iCardio is available online and is open-access at the National Institute of Science and Technology for Health Technology Assessment (Instituto Nacional de Ciência e Tecnologia para Avaliação de Tecnologias em Saúde) website.^6^

Indicators are based on data from 291,490 patients with 317,338 index hospitalizations (172,312 in 2019 and 149,026 in 2020), and 375,809 procedures (172,874 of cardiovascular operations and 202,935 of interventional cardiology) performed in 558 health care centers.

The indicators are presented in 4 different views: “patients’ profile,” “by location,” “procedure rates,” and “detailed exploration.” The data are presented by year, and, in each visualization, it is possible to select the indicator of interest and apply and combine a variety of filters including patients’ and hospitalizations’ characteristics. The combination of different indicators and the application of filters can be used to overview advanced cardiovascular care in Brazil, exploring geographic differences at different levels, including regions, federative units, municipalities, and even individual health care centers. Figure 1 presents a view of the dashboard delimiting the main features. Geographic variation for primary percutaneous coronary intervention rates is shown in Figure 2.

**Figure 1:**
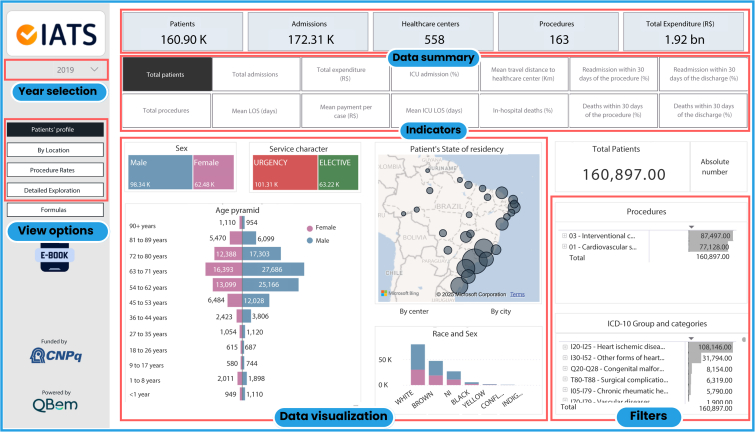
iCardio cover visualization and main features.Red boxes and tags are added to depict the main features of the tool. This figure presents a translated image, which is available in Portuguese.

**Figure 2:**
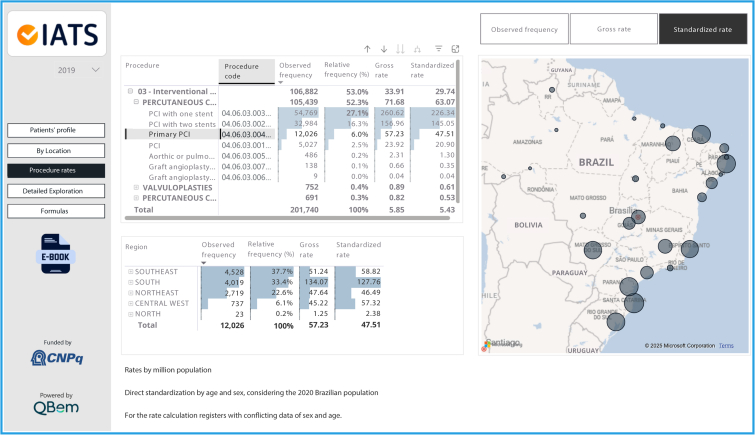
Geographic variation of primary percutaneous coronary intervention in SUS, shown in iCardio “Procedure rates” view. Circles in the Brazilian map represent the differences in the standardized procedure rates in each Federative Unit. The upper table shows the frequencies and rates by procedure, allowing the selection of the specific procedure of interest and the lower table shows the frequencies and rates of the selected procedure by geographic Region. This figure presents a translated image of the tool, which is available in Portuguese.

### Application

Developing solutions with intelligence to manage specific procedures at a patient and center level is paramount to making a patient-centered management process feasible in the health care system. This study innovates by introducing an interactive web platform containing RWD to evaluate disparities, resource consumption, and equity indicators of cardiovascular diseases across the Brazilian states. It represents a transformative tool for improving efficiency at the biggest universal health care system, as the information available can serve as a source for analysis driven to enhance the quality of care, reduce variability, and inefficiency of the health care system in Brazil.

This platform is available to all stakeholders—clinicians, hospital managers, payers, industry, and the general population—and can be used freely for research or management purposes. Meaningful and accessible RWD facilitates data reuse, which is a stepping stone to the emergent third wave of open data,^7^ potentially accelerating research and improving evidence generation and decision-making efficiency, supporting Health Economics, Outcomes Research, Policy Making, and Clinical Trial Designs.^8^, ^9^, ^10^, ^11^

The use of RWD by policymakers and health care leaders has increased exponentially in the last few years,^12^ targeting centralizing care on patients’ needs, reducing waste, and increasing value in health care systems.^13^ Because of the opportunity to make accurate information transparent for all stakeholders, incorporating data-driven and more effective policies has received great potential in Brazil, which is scarce yet in most Latin American countries.^1^^,^^14^, ^15^, ^16^ However, to achieve that goal, it is necessary to replicate the efforts added to create the first platform for cardiovascular procedures to other priority fields in the health care systems, such as oncological care.

### Limitations

The platform was developed as part of a research project and is not a solution implemented within the health system's management tools. Currently, it is only available in Portuguese. In addition, our analysis is subject to the traditional risks associated with using linkage data, including missing information and misclassification, as well as any updates on the platform being dependent on the continued data linkage process by Departamento de Informática do SUS.

## Conclusion

The iCardio is a population RWD-based platform that provides access to relevant information about cardiovascular care in Brazil for all stakeholders, including policymakers, clinicians, payers, providers, and patients. Because of its level of granularity, the information generated can serve as a transformative tool in guiding policymakers’ informed decisions and answering specific research questions based on population-based data.

## Potential Competing Interest

The authors report no competing interests.

## References

[1] Brazil. Ministério da Saúde. Secretaria Executiva Departamento de Monitoramento, Avaliação e Disseminação de Dados e Informações Estratégicas em Saúde - DEMAS. Plano de Dados Abertos para o Ministério da Saúde - 2022-2023. https://www.gov.br/saude/pt-br/acesso-a-informacao/dados-abertos/pda/plano-de-dados-abertos_ms_2022-2023.pdf/view.

[2] Guerra Junior A.A., Pereira R.G., Gurgel E.I. (2018). Building the national database of health centred on the individual: administrative and epidemiological record linkage - Brazil, 2000-2015. Int J Popul Data Sci.

[3] da Silva Etges A.P.B., Nabi J., Geubelle A., Martins S.O., Polanczyk C.A. (2022). Analytical solutions to support value-based health care: the ischemic stroke care pathway case. NEJM Catal Innov Care Deliv.

[4] Oliveira G.M.M., Brant L.C.C., Polanczyk C.A. (2022). Cardiovascular Statistics - Brazil 2021. Arq Bras Cardiol.

[5] Brazil. Ministério da Saúde DATASUS. Sistema de Gerenciamento da Tabela de Procedimentos, Medicamentos, Órteses, Próteses e Materiais Especiais (SIGTAP) do SUS. Brasília2024. http://sigtap.datasus.gov.br/tabela-unificada/app/sec/inicio.jsp.

[6] Instituto Nacional de Ciência e Tecnologia para Avaliação de Tecnologias em Saúde - INCT/IATS [National Institute of Science and Technology for Health Technology Assessment] “iCardio: Painel de Indicadores em Atenção Cardiovascular no SUS” [“iCardio: Dashboard of Brazil's universal healthcare system cardiovascular care indicators”]. https://www.iats.com.br/icardio.

[7] Verhulst S., Young A., Zahuranec A.J., Calderon A., Gee M., Aaronson S.A. (2020). The emergence of a third wave of open data: How to accelerate the re-use of data for public interest purposes while ensuring data rights and community flourishing. SSRN Journal.

[8] Dagenais S., Russo L., Madsen A., Webster J., Becnel L. (2022). Use of real-world evidence to drive drug development strategy and inform clinical trial design. Clin Pharmacol Ther.

[9] Magalhães T., Dinis-Oliveira R.J., Taveira-Gomes T. (2022). Digital health and big data analytics: implications of real-world evidence for clinicians and policymakers. Int J Environ Res Public Health.

[10] Gomes M.A.S., Kovaleski J.L., Pagani R.N., da Silva V.L., Pasquini T.C.S. (2023). Transforming healthcare with big data analytics: technologies, techniques and prospects. J Med Eng Technol.

[11] Lee W.C. (2024). Seeing the whole elephant: integrated advanced data analytics in support of RWE for the development and use of innovative pharmaceuticals. Expert Rev Pharmacoecon Outcomes Res.

[12] Basch E., Schrag D. (2019). The evolving uses of “real-world” data. JAMA.

[13] Sherman R.E., Anderson S.A., Dal Pan G.J. (2016). Real-world evidence - what is it and what can it tell us?. N Engl J Med.

[14] Brazil. Agência Nacional de Vigilância Sanitária - Anvisa (2023).

[15] Center for Strategic Studies and Management (2022).

[16] Brasília: Ministry of Health of Brazil 1º Relatório de Monitoramento e Avaliação da Estratégia de Saúde Digital para o Brasil 2020-2028 [Brazil. Ministry of Health of Brazil. Executive Secretariat. Department of Informatics of the Brazilian NHS. 1st Brazilian National Digital Health Strategy 2020-2028 Monitoring and Evaluation Report/Ministry of Health of Brazil, Executive Secretariat, Department of Informatics of the Brazilian NHS; 2021:83. http://bvsms.saude.gov.br/bvs/publicacoes/1st_brazilian_national_digital_health_strategy.pdf.

